# Interplay between Zn^2+^ Homeostasis and Mitochondrial Functions in Cardiovascular Diseases and Heart Ageing

**DOI:** 10.3390/ijms23136890

**Published:** 2022-06-21

**Authors:** Siarhei A. Dabravolski, Nikolay K. Sadykhov, Andrey G. Kartuesov, Evgeny E. Borisov, Vasily N. Sukhorukov, Alexander N. Orekhov

**Affiliations:** 1 Department of Clinical Diagnostics, Vitebsk State Academy of Veterinary Medicine [UO VGAVM], 210026 Vitebsk, Belarus; 2 Laboratory of Angiopathology, Institute of General Pathology and Pathophysiology, Russian Academy of Medical Sciences, 125315 Moscow, Russia; drawnman@mail.ru (N.K.S.); andkartuesv@gmail.com (A.G.K.); 3 Petrovsky National Research Centre of Surgery, 2, Abrikosovsky Lane, 119991 Moscow, Russia; borisovevgenij5@gmail.com (E.E.B.); vnsukhorukov@gmail.com (V.N.S.); 4 Institute for Atherosclerosis Research, 121609 Moscow, Russia; a.h.opexob@gmail.com

**Keywords:** zinc homeostasis, ER stress, mitophagy, cardiovascular disease, ageing

## Abstract

Zinc plays an important role in cardiomyocytes, where it exists in bound and histochemically reactive labile Zn^2+^ forms. Although Zn^2+^ concentration is under tight control through several Zn^2+^-transporters, its concentration and intracellular distribution may vary during normal cardiac function and pathological conditions, when the protein levels and efficacy of Zn^2+^ transporters can lead to zinc re-distribution among organelles in cardiomyocytes. Such dysregulation of cellular Zn^2+^ homeostasis leads to mitochondrial and ER stresses, and interrupts normal ER/mitochondria cross-talk and mitophagy, which subsequently, result in increased ROS production and dysregulated metabolic function. Besides cardiac structural and functional defects, insufficient Zn^2+^ supply was associated with heart development abnormalities, induction and progression of cardiovascular diseases, resulting in accelerated cardiac ageing. In the present review, we summarize the recently identified connections between cellular and mitochondrial Zn^2+^ homeostasis, ER stress and mitophagy in heart development, excitation–contraction coupling, heart failure and ischemia/reperfusion injury. Additionally, we discuss the role of Zn^2+^ in accelerated heart ageing and ageing-associated rise of mitochondrial ROS and cardiomyocyte dysfunction.

## 1. Introduction

Zinc is a redox inactive element, a crucial component of the antioxidant defence system, maintaining the cell redox balance, and is involved in a multitude of physiological functions [1]. In many cells and tissues, labile Zn^2+^ plays an important role as a signalling molecule, structural component or major regulator of macromolecules; therefore, even mild disturbance in Zn^2+^ homeostasis may impact human health, lead to neurodegenerative and neurodevelopmental disorders, diabetes and obesity, malfunction of reproductive system, cardiovascular diseases and other pathological conditions [2,3]. In particular, a high intracellular labile Zn^2+^ ([Zn^2+^]_i_) level is toxic for cardiomyocytes, where [Zn^2+^]_i_ also takes part in excitation–contraction coupling and in excitation–transcription coupling [4]. Mammalian cells have different ways to regulate [Zn^2+^]_i_ homeostasis. Zinc could be released from metalloenzymes or metalloproteins, several other intracellular storages and organelles, regulated through Zn^2+^-binding molecules and Zn^2+^ sensors, and transported by the two main Zn^2+^ transporter families Zrt-/Irt-like protein (ZIP) and zinc transporters (ZnT) [5].

It is known that the development of cardiac dysfunction is closely associated with increased mitochondrial reactive oxygen species (ROS) production, which could be caused by different pathological conditions (such as hyperglycaemia or hyperlipidaemia). In particular, Zn^2+^-associated signalling pathways are involved in the development of diabetic cardiomyopathy and other heart diseases [6,7]. In that regard, the close coordination of [Ca^2+^]_i_ and [Zn^2+^]_i_ homeostasis in mitochondrion balances the redox status and oxidative stress status of cardiomyocytes and provides a cardioprotective effect [8].

A number of studies suggest that both Zn^2+^ deficiency and excess zinc are harmful to cells, leading to impaired excitation–contraction cycling in cardiomyocytes, metabolic disorders and growth retardation [9,10]. Under normal physiological conditions, the cytosolic labile [Zn^2+^]_i_ in cardiomyocytes is less than 1 nM, while it could increase ~2 fold under chronic hyperglycaemia conditions or even ~30-fold under acute oxidant exposure, although total cellular Zn^2+^ is about 200 µM [11,12]. Additionally, mitochondria were proved to be another crucial intracellular Zn^2+^-pool in cardiomyocytes, thus, further connecting mitochondria as a major cellular ROS producer, Zn^2+^-mediated alterations in mitochondria ultrastructure and their role in the pathogenesis of many cardiovascular diseases [13,14]. The role of Zn^2+^ homeostasis in diabetes mellitus and obesity-induced cardiac inflammation, remodelling, dysfunction and other cardiovascular diseases and complications was thoroughly covered in several recent excellent reviews [9,15,16]. Therefore, those topics will be excluded from our review. We wish to redirect interested readers to the suggested papers.

## 2. Zn^2+^ in Mitochondrial Homeostasis

Although mitochondria represent an important intracellular Zn^2+^ pool and both insufficient or excessive Zn^2+^ are deleterious to the mitochondrial structure and function, it is not known to date how Zn^2+^ is transported into and out of mitochondria. Surplus Zn^2+^ accumulation in mitochondria has diverse negative effects on the mitochondria, where it inhibits α-ketoglutarate dehydrogenase and complexes I and III of the electron transport chain (ETC), increases ROS production and causes loss of MMP (mitochondrial membrane potential) and activates mPTP (mitochondrial permeability transition pore), which leads to the release of pro-apoptotic factors and cell death [17]. Further, zinc overload causes abnormal morphological changes: mitochondria had shortened in length and increased in circularity, decreased in major axis and in aspect ratio [18]. Incubation of cardiomyocytes with a Zn^2+^ source (as Zn^2+^ pyrithione) increases the phosphorylation levels of several signalling proteins, such as Glycogen Synthase Kinase-3 Beta (GSK3β), Protein Kinase B (Akt) and nuclear factor-κ B (NFκB), and increases the level of endoplasmic reticulum (ER) stress proteins, such as Heat Shock Protein 70 Family Protein 5 (GRP78) and calregulin (Endoplasmic Reticulum Resident Protein 60) and ER-mitochondria contact sites protein promyelocytic leukemia protein (PML), thus, suggesting the involvement of Zn^2+^ in ER-mitochondria cross-talk and ER stress [19]. 

Zn^2+^ is involved in the regulation of mitochondrial dynamics (fission and fusion) and mitophagy, a quality control mechanism, specialised form of autophagy, targeted to degrade damaged and superfluous mitochondria to reinstate cellular homeostasis in response to various stresses. Mitochondrial fission is regulated by *DRP1* (DNM1L or dynamin 1 like)/*FIS1* (fission, mitochondrial 1) and fusion is regulated by *MFN1*, *MFN2* (mitofusin 1 and 2)/*OPA1* (optic atrophy protein 1) genes [20]. While there are several defined mitophagy mechanisms, in general, they could be divided into two main types: conventional PTEN-Induced Kinase 1/Parkin RBR E3 Ubiquitin Protein Ligase (PINK1/Parkin)-mediated and alternative Parkin-independent mitophagy [21,22]. After damaged parts of mitochondria are digested during mitophagy, healthy parts of mitochondria are fused back to the mitochondrial network and continue normal functioning [23]. Mitophagy and balanced fission/fusion are crucial for many cellular processes, normal organism development and functioning, and their dysregulation is associated with many developmental and neurodegenerative diseases, cancer, inflammageing and other pathologies [24,25]. As it was found, ROS promotes the release of lysosomal Zn^2+^ and increases mitochondrial Zn^2+^, which triggers mitochondrial division by promoting mitochondrial recruitment of Drp1 [26]. Further, a Drp1–ZIP1 interaction could stimulate Zn^2+^ entry into mitochondria, thus, reducing the MMP and promoting mitochondrial fission and mitophagy [27]. Furthermore, high mitochondrial Zn^2+^ might promote PINK/Parkin-mediated mitophagy by increasing *BECN1* (Beclin1 or ATG6, Autophagy Related) expression in cardiomyocytes in response to hypoxia oxygenation (H/R) [28].

A recent study on the Caenorhabditis elegans model suggested that Znt9 (SLC-30A9, Solute Carrier Family 30 Member 9) is the mitochondrial Zn^2+^ exporter, the loss of which causes Zn^2+^ accumulation in mitochondria and dysregulation of the mitochondrial structure and functions, aggravating normal animal development and reducing lifespan. Moreover, SLC-25A25/SCaMC-2 was identified as an important regulator of mitochondrial Zn^2+^ import, the losing of which suppresses functional and structural defects caused by a loss of SLC-30A9. It was shown that mitochondria mostly import Zn^2+^ from the endoplasmic reticulum Zn^2+^ pool [29]. Similarly, the role of SLC-30A9 in Zn^2+^ export from mitochondria was also confirmed in humans, where loss of SLC-30A9 leads to excessive Zn^2+^ accumulation in mitochondria, severe mitochondrial swelling, increased ROS production and dysregulated metabolic function. Additionally, it was proposed that SLC-30A9 is crucial for sperm activation and organismal fertility, and in neurons, the SLC-30A9 mutation is responsible for Birk–Landau–Perez cerebrorenal syndrome—an autosomal recessive syndrome, characterized by nephropathy, muscle weakness, intellectual disability, camptocormia and oculomotor apraxia [30,31].

In summary, the dysregulation of cellular Zn^2+^ homeostasis leads to mitochondrial and ER stresses, interrupts normal ER/mitochondria cross-talk and mitophagy, and subsequently, results in increased ROS production and dysregulated metabolic function. Besides cardiac structural and functional defects, insufficient Zn^2+^ transport was associated with development abnormalities, such as Birk–Landau–Perez cerebrorenal syndrome. Therefore, further investigation of Zn^2+^-regulating treatments to prevent mitochondrial zinc overload and the associated changes in mitochondrial functions could provide vital mechanistic insights for understanding and treating these human diseases.

## 3. Zn^2+^ in Cardiac Function and Pathology

Impaired Zn^2+^ homeostasis is associated with developmental defects, including multiple types of cardiac abnormalities, a variety of cardiovascular disorders and diseases. Sufficient Zn^2+^ supply is crucial for normal heart electrical and mechanical functions and the operation of redox signalling pathways. However, Zn^2+^ levels beyond a narrow concentration range are toxic and damaging for cardiomyocytes [32]. Further, in this section, we discuss the role of Zn^2+^ in heart development, cardiac contraction and arrhythmia, heart failure, ischaemia/reperfusion injury and heart ageing.

### 3.1. Zn^2+^ in Heart Development

Heart development is a sophisticated process orchestrated by multiple transcription factor families, which regulate myocardial gene expression, vessel and muscle growth, and morphogenesis, which eventually result in the formation of a functionally competent ventricle wall. In the first step, the tubular heart is composed of an outer one-cell layer of myocardium and an inner one-cell layer of endocardium separated by cardiac jelly (or ECM—extracellular matrix). In the next step (trabeculation), cardiomyocytes protrude into the cardiac jelly and form rapidly growing structures—trabeculae. Trabeculae facilitate nutrient and oxygen exchange between the blood and the heart before coronary circulation is arranged. Accompanying the initiation of coronary circulation, trabeculae go through the compaction process, collapsing into the ventricle wall and becoming part of the compact myocardium. In a further stage, the ventricle wall has a thick compact myocardium composed of multi-layered spiral cardiomyocytes, with few trabeculae on the surface [33].

Zinc is an important microelement, involved in gene transcription, protein translation and maintaining the structural stability of organelles via synthesis and activity regulation of various metalloenzymes, transcription factors and RNA polymerase [34]. Recent research suggested that gestational zinc deficiency is a cause of congenital heart disease (CHD) in a mouse foetus [35]. CHD combines a wide range of birth defects, which are present from birth and affect the normal way the heart works. It was established that zinc deficiency induced *SENP5* overexpression, which led to cardiac dysplasia. SUMO1/Sentrin Specific Peptidase 5 (SENP5) is responsible for the reversible posttranslational modification of proteins by the addition of small ubiquitin-like SUMO proteins, altering the conformation, localization and stability of the target protein, and is involved in regulation of the ribose biosynthesis, stem cell maintenance and differentiation, cell cycle, DNA damage repair, chromatin remodelling and gene transcription [36]. In-vitro experiments on cardiomyocytes differentiated from human-induced pluripotent stem cells under a zinc deficiency condition showed increased *SENP5* expression, reducing conjugated SUMO during heart development and causing increased cell apoptosis and decreased viability, and further resulted in myocardial abnormality [35].

Recent research proposed the cellular zinc importer ZIP8 (Solute carrier family 39 member 8 or Slc39a8) as a novel regulator of ventricular myocardial development [37]. Left ventricular noncompaction (LVNC) is a form of CHD caused by arrested compaction, which usually affects both ventricles and is characterized by excessive trabeculation with deep intertrabecular recesses and a thin compact myocardium. Typical LVNC complications are systemic embolic events, ventricular arrhythmias and heart failure [38,39]. Homozygous Slc39a8-null mice embryos do not survive embryogenesis and exhibit an LVNC-similar cardiac phenotype. It was shown that *SLC39A8* is expressed by endothelial cells in the developing mouse heart, where it is involved in ECM degradation through the MTF1 (metal regulatory transcription factor 1), which promotes the expression of several ECM-degrading A Disintegrin and Metalloproteinase with Thrombospondin Motifs 1 (ADAMTS) metalloproteinases. Therefore, SLC39A8 knockdown decreases cellular Zn uptake, with a subsequent reduction in MTF1 transcription activity, expression of Adamts metalloproteinases and impaired cardiac ECM degradation [37].

### 3.2. Zn^2+^ in Excitation–Contraction Coupling

Molecular and pharmacological studies have shown that labile Zn^2+^ can permeate membranes through both ligand-gated channels and voltage-activated Ca^2+^ channels, and also significantly affect the functionality of these channels. [Zn^2+^]_i_ is widely recognized as a vital intracellular second messenger, known to regulate K^+^ ATP-channels (ATP-dependent K^+^-channels), M-type K^+^-channels, both L-type and T-type of Ca^2+^-channel currents and Cl^−^-conductance, thus, affecting the electrical activity of a single cardiomyocyte and further stimulating an induction of arrhythmia at the cellular level [40]. Additionally, an increase in [Zn^2+^]_i_ levels is closely associated with increased production of ROS/reactive nitrogen species (RNS) and induction of thiol oxidation and hyper-phosphorylation of many cellular proteins and kinases, thus, affecting the contractile machinery of cardiomyocytes [32].

Recent research suggested that high [Zn^2+^]_i_ could inhibit voltage-dependent K^+^-currents, thereby altering cardiac function by prolonging action potential. In particular, a high [Zn^2+^]_i_ level significantly lowers the cellular ATP level, inhibits transient outward K^+^-currents, steady-state currents and inward-rectifier K^+^-currents, leading to a modulation of depolarization in resting membrane potential, prolongations in action potential repolarizing phases and induction of spontaneous action potentials [41].

Overall, the presented data suggested a crucial role for the high cardiomyocytes [Zn^2+^]_i_ level in the induction of cardiac contraction and arrhythmia. Therefore, application of [Zn^2+^]_i_ controlling drugs/compounds could be a good candidate for a novel therapeutic target in cardiac dysfunction. However, further studies to investigate the underlying physiology of [Zn^2+^]_i_ action in cardiomyocytes are required.

### 3.3. Zn^2+^ in Heart Failure

Heart failure (HF) is a clinical syndrome of progressive functional degradation when cardiac output is inadequate to meet the needs of the organism. HF may be present from birth (congenital heart disease) or occur secondarily, because of various insults, such as atherosclerosis, myocardial infarction, hypertension, arrhythmia, toxic stimuli and others, affecting over 64 million people worldwide [42,43]. Mitochondrial dysfunction, including defects in mitophagy, redox signalling, biogenesis, oxidative phosphorylation, network organization, unfolded protein response, fission/fusion and substrate selection, are widely recognised as significant factors in HF progression [44]. Recent research shows that increased mitochondrial ROS is the main driver of both acute events (such as electrical instability, responsible for sudden cardiac death) and chronic HF-associated remodelling events (disordered expression and phosphorylation of proteins crucial for excitation–contraction and antioxidant defence system) [45].

Experimental and clinical evidence suggests a crucial role for the crosstalk between Zn^2+^ and Ca^2+^ transporters and channels in the control of cardiac contractility. In particular, the cardiac ryanodine receptor (RYR2), known to conduct Ca^2+^ in vivo and other divalent cations in vitro, has attracted much attention [7]. During the cardiac cycle, myocardial contraction is initiated by Ca^2+^ influx into the cell, where it binds to and activates the RyR2 receptor. Further, the opening of RyR2 channels releases Ca^2+^ from the sarcoplasmic reticulum (SR), which results in a transient rise in cytosolic Ca^2+^. Because of the combined action of RyR2 channel closure, extrusion of Ca^2+^ from the cell and uptake of Ca^2+^ back into the SR Ca^2+^ stores, the Ca^2+^ concentration is reduced and causes cardiac muscle relaxation. The current understanding suggests that HF RyR2 channels cannot remain closed during diastole and become “leaky”, leading to dysregulated Ca^2+^ homeostasis in cardiomyocyte and spontaneous Ca^2+^ spark frequency, resulting in irregular contractile activity and decreased systolic contraction. Additionally, there is some evidence for the involvement of unknown channels, facilitating an RyR2-independent mechanism of Ca^2+^ efflux [46].

Indeed, recent research suggested that pathophysiological concentrations of Zn^2+^ not only dysregulated RyR2-channel openings but also activated the transmembrane protein mitsugumin 23 (MG23) [47], a recently identified voltage-dependent non-selective cation-conducting channel abundant in the ER/SR, where it was proposed to participate in regulating Ca^2+^ dynamics alongside RyR2 [48].

Additionally, in HF, increased [Zn^2+^]_i_ induces ER stress via increased total PKC, *PKCα* expression and PKCα-phosphorylation. Increased levels of [Zn^2+^]_i_ were associated with significantly elevated levels of ER stress markers GRP78, CHOP/Growth Arrest and DNA Damage-Inducible Protein GADD153 (Gadd153) and calnexin. Further, HF was associated with increased expression of *ZIP14* and *ZNT8* and decreased levels of *ZIP8* Zn^2+^ transporters [49]. Interestingly, that activated PKCα is known as an important mediator of induction of ventricular arrhythmias [50] and effector of oxidative tissue injury [51]. Similarly, experiments on a transverse aortic constriction (TAC) rat model, mimicking the development of cardiac hypertrophy-associated heart failure, demonstrated increased expression of ER stress markers (*GRP78*, *CHOP*/Gadd153 and calnexin) and apoptotic status markers (Glycogen Synthase Kinase-3 Beta (*GSK3B*), Bax-to-Bcl-2 ratio and BCL2-Binding Component 3 (*BBC3*)). Further, the ratios of phosphorylated to non-phosphorylated Akt, NFκB and PKCα proteins were significantly higher in the TAC group. Heart tissue from the TAC group had increased expression levels of *ZIP7*, *ZIP14* and *ZNT8* transporters, while the expression levels of *ZIP8* and *ZNT7* were decreased [52]. Recent research has identified the involvement of SLC39A2 (ZIP2) in the development of cardiac hypertrophy and heart failure [53]. Interestingly, ZIP2 knockdown enhances innate immune signalling pathways (NFκB, TOLL-like receptor and interferon regulatory factors (IRFs)), which are responsible for programmed cell death, inflammatory response and defence against microbes [54,55,56], while the expression of IκBα (Inhibitor of NFκBα) was reduced. Those data demonstrate the tight regulatory circuit between ZIP2-mediated zinc homeostasis and remodelling of innate immune signalling in cardiomyocyte hypertrophy [53].

Interestingly, zinc deficiency (caused by malnutrition, intestinal malabsorption or other reasons) is associated with the development and deterioration of HF via several pathobiological pathways (systemic inflammation, dysregulated apoptosis and oxidative stress) [57]. As it was shown on hearts of weaned piglets affected by subclinical zinc deficiency, the level of glutathione and the expression of ROS-detoxifying enzymes (glutathione, glutathione reductase, catalase and metallothionein 1A) was decreased, while the expression of pro-apoptotic genes (B-cell lymphoma 2–associated X protein (*BAX*) and caspase 9 (*CASP9*)) was increased and the expression of (*FAS*), etoposide-induced 2.4 (*EI24*) and cyclin-dependent kinase inhibitor 1A (*CDKN1A*) correlated positively to cardiac zinc level in piglets [12]. Recent clinical data clearly support such an association, suggesting to use zinc level as a possible biomarker of cardiovascular health and application of zinc supplementation on outcomes in patients with HF [58,59].

In conclusion, zinc deficiency is linked to the development and progression of HF syndromes, where [Zn^2+^]_i_ (controlled via Zn^2+^ transporters) acts on the intersection between ER stress and PKCα activation pathways in HF induction. The number of available studies suggests the high therapeutic potential of zinc supplementation, which, however, requires further clinical confirmation, ideally, in randomized trials.

### 3.4. Zn^2+^ in Ischemia/Reperfusion Injury

Myocardial ischemia/reperfusion injury (I/R) is a very common cardiovascular disease with a high mortality rate, which is caused by an inadequate supply to the heart of blood and nutrients, because of a narrowing or closure of the coronary arteries or increased myocardial substrate demand. Pathogenesis of myocardial infarction is caused by intracellular Ca^2+^ overload, ATP depletion, increased migration of neutrophils to the ischemic tissue, acidosis, ROS overproduction and activation of inflammatory, ER stress, apoptosis and other pathways [60,61]. Recent research suggests protective effects from Zn^2+^ against I/R through stimulation of anti-oxidant defence, anti-inflammatory and other mechanisms, thus, reducing ischemic injury and facilitating recovery [62]. Further in this section, we cover recent research explaining the role of Zn^2+^ in ischemia/reperfusion injury and the related in-vitro model (hypoxia–reoxygenation injury).

#### 3.4.1. Mitochondria as the Main Source of Chemically Reactive Species in I/R Injury

Chemically reactive species are a product of both normal cellular physiological processes and pathological conditions and are usually represented by oxygen and/or nitrogen (reactive oxygen and nitrogen species). In general, increased ROS and/or RNS has been associated with many cardiovascular diseases, including ischaemia/reperfusion injury. Imbalance between ROS/RNS generation and removal causes oxidative/nitrosative alterations of proteins, lipids, carbohydrates and nucleic acids, which result in derangements of cellular structures, transduction of hormonal stimuli, inflammatory and apoptosis signalling [63]. Because mitochondria occupy a significant volume of cardiomyocytes and utilize more than 90% of oxygen reaching the cardiac muscle, it is not surprising that a substantial part of ROS generation occurs in mitochondria. For this reason, mitochondria are the main targets of many signalling pathways involved in different cardioprotection strategies [64]. 

**Figure 1:**
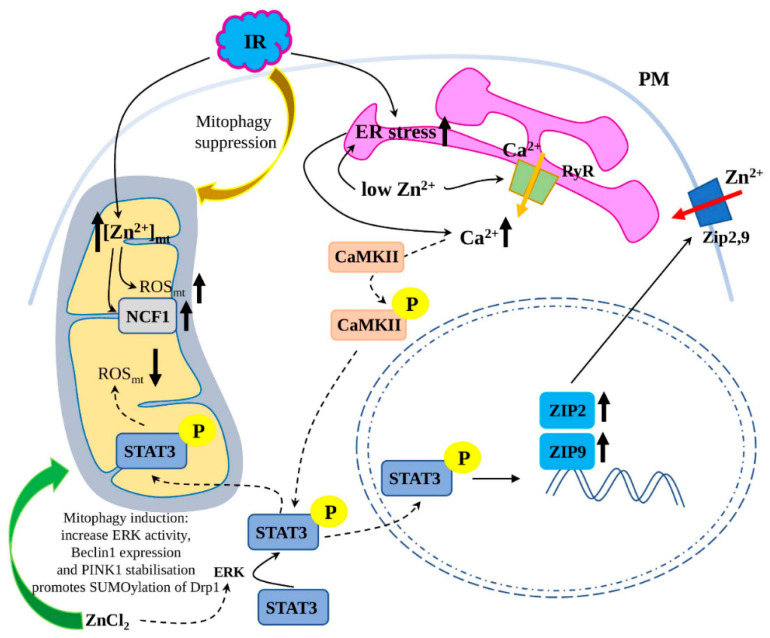
The role of Zn^2+^ in ischemia/reperfusion injury (I/R). I/R was shown to suppress mitophagy, increase mitochondrial reactive oxygen species (ROS) production and induce endoplasmic reticulum (ER) stress. I/R leads to increased Zn^2+^ accumulation in mitochondria, further increasing ROS production via (Neutrophil Cytosolic Factor 1) NCF1. I/R, similarly to Zn^2+^ deficiency, induces ER stress, which leads to ryanodine receptor (RyR) channel activation and increase in intracellular Ca^2+^ level and Ca^2+^-calmodulin-dependent protein kinase (CaMKII) phosphorylation, which subsequently acts on Signal Transducer and Activator of Transcription 3 (STAT3) to stimulate *ZIP2* and 9 expression. Zn^2+^ treatment provides cardioprotective effect by stabilising PTEN Induced Kinase 1 (PINK1) and dynamin 1 like (Drp1) proteins, increasing ATG6, Autophagy Related (Beclin1) expression and ERK activity, thus stimulating mitophagy. Additionally, ERK phosphorylates STAT3 in a Zn^2+^-dependent way. The direction of Zn^2+^ transport is depicted with a red arrow, Ca^2+^—orange, increased/decreased levels of metabolites/gene expression—black; PM—plasma membrane.

As it was shown on the HeLa cells, subjected to chemical ischaemia, stimulated by 30 min of oxygen and glucose deprivation, Zn^2+^ is released from intracellular stores and accumulated in mitochondria (Figure 1). Further, a rise in Zn^2+^ was followed by a rise in mitochondrial ROS production and congruent with an increase in the functional component of NADPH oxidase, p47phox (NCF1) [65], which produces superoxide anion [66]. Thus, a positive feedback loop during ischemic stress is suggested, where an excess of free Zn^2+^ and mitochondrial ROS closely connected via NADPH component p47phox [65].

Myocardial zinc homeostasis contributes to the cardioprotective effect from hypoxia/reoxygenation injury, ischemia/reperfusion injury and ischemia postconditioning. One of the Zn^2+^-mediated cardioprotective mechanisms relies on the activation of STAT3 (Figure 1), which is phosphorylated at Ser727 by Mitogen-Activated Protein Kinase 1 or ERK (MAPK1) and important for regulating cardioprotective proteins, mPTP opening, mitochondrial respiration and ETC activity [67,68]. As it was shown on mitochondria isolated from rat hearts subjected to reperfusion, ZnCl_2_ treatment increases the level of phospho-Ser727 STAT3 in mitochondria, which improves the mitochondrial oxidative phosphorylation and increases the mRNA level of the complex I subunit ND6, reduces mitochondrial ROS generation and preserves mitochondrial membrane potential (ΔΨm) [69].

Interestingly, another study demonstrated that *ZIP2* expression was increased in an STAT3-dependent way at reperfusion in in-vivo mouse hearts in an attempt to compensate Zn^2+^ loss (Figure 1). Knockout of Zip2 genes (Zip2^+/−^ and Zip2^−/−^) aggravates I/R injury, and STAT3 gene delivery failed to reduce I/R injury in Zip2^−/−^ mice, while the delivery of both genes (Zip2 and STAT3) to wild-type mice reduced I/R injury [70].

#### 3.4.2. Zn^2+^ Regulation of Mitophagy in I/R Injury

An increasing body of evidence shows a vital role for Zn^2+^ in the regulation of autophagy and cardiac homeostasis. Mitophagy, a specialised form of autophagy, selectively targets and degrades damaged or dysfunctional mitochondria, thus, preventing excessive production of ROS and playing a cardioprotective function in a wide range of cardiac diseases [71,72]. As it was shown on cardiac myoblasts (H9c2 cell line) subjected to hypoxia/reoxygenation, zinc treatment (in form of ZnCl_2_) induces autophagy and mitophagy by increasing ERK activity, Beclin1 expression and stabilising PINK1 (Figure 1). At the reoxygenation stage, those Zn^2+^-mediated effects prevented mitochondrial superoxide generation and dissipation of mitochondrial membrane potential (ΔΨm), thus, protecting the heart from H/R injury [28].

Similarly, recent research has shown that zinc regulates mitophagy and contributes to the cardioprotection by modulating the small ubiquitin-like modifier (SUMO) system, which is responsible for the involvement of modified proteins in different cellular processes (apoptosis, transcriptional regulation, protein stability and nuclear transport) [73]. Zn^2+^ treatment reverses the negative H/R effects (decreased SUMO1 modification level of proteins and increased myocardial apoptosis) and promotes SUMOylation of Drp1, the crucial regulator of mitophagy [74].

Myocardial reperfusion injury upregulates mitochondrial Zn^2+^ transporter ZIP7 and suppresses mitophagy [75]. As it was shown on mouse hearts and human heart samples with the cardiac-specific ZIP7 conditional knockout and subjected to ischemia/reperfusion in vivo, ZIP7 is upregulated at reperfusion, which leads to mitochondrial hyperpolarization and prevents PINK1 and Parkin accumulation in mitochondria, thus, suppressing mitophagy. Similarly, ZIP7 is markedly upregulated in cardiac mitochondria samples from patients with heart failure. On the contrary, ZIP7 knockout enhanced mitophagy upon reperfusion, reduced mitochondrial ROS generation and myocardial infarction size in a PINK1-dependent manner [75]. Therefore, timely normalisation of the mitochondrial Zn^2+^ level via downregulation or inactivation of the mitochondrial *ZIP7* expression could be beneficial for patients with acute myocardial infarction and other cardiovascular diseases. Further, because impaired mitophagy is associated with many other diseases and disorders (such as Alzheimer’s and Parkinson’s diseases, premature ageing, atherosclerosis and others) [76,77], zinc-based modulation of the mitophagy rate with therapeutic or pharmacological approaches may have wide clinical applications.

#### 3.4.3. Zn^2+^-Mediated Regulation of ER Stress in I/R Injury

ER is responsible for various cellular functions, including protein production and folding, regulation of Ca^2+^ levels, steroids and lipid synthesis and metabolism. However, different cellular stresses could interrupt normal protein synthesis, disturb proper protein folding or cause accumulation of non-properly folded proteins and activated so-called unfolded protein response (URP). Prolonged activation of the UPR disrupts normal ER functions and leads to an “ER stress” state, which is accompanied by the activation of various ER stress chaperones or proteins, such as calregulin, GRP78 and GRP94. Multiple studies have proved the close association of ER stress with I/R injury and other cardiovascular diseases (reviewed in [78,79]).

Interestingly, a recent report suggested that reperfusion initiates ER stress but not ischaemia. Analysis of *GRP78* and *GRP94* expression during ischaemia and reperfusion in combination with ER stress inhibitor tauroursodeoxycholic acid (TUDCA) and zinc chelator *N*,*N*,*N*′,*N*′-tetrakis-(2-pyridylmethyl) ethylenediamine (TREN) proved involvement of Zn^2+^ in ER stress inhibition-induced cardioprotection against I/R injury by modulation of the mPTP opening. Application of TUDCA increases intracellular free zinc, reduces infarct size, prevents ER and mitochondrial damages at reperfusion, thus, protecting the heart from reperfusion injury in a Zn^2+^-dependent way [80].

The treatment of cardiomyocytes with zinc pyrithione provides several beneficial effects after H/R; in particular, it normalises depleted intracellular zinc, enhances intracellular superoxide production, mitigates H/R-induced UPR and improves proteasomal function. Further, zinc pyrithione suppresses NADPH oxidase 2 (NOX2), which is responsible for ROS production and promotes Erb-B2 Receptor Tyrosine Kinase 2 (ErbB2) signalling, thus, improving cell survival after H/R [81]. The dysregulation of ErbB2 expression was associated with many cancer types, heart failure and ischemic heart disease; ErbB dimerization and activation of PI3K/Akt cascade suppresses cardiomyocyte cell death through caspase inactivation [82]. Thus, Zn^2+^ replenishment after myocardial H/R alleviates associated oxidative stress, UPR and improves cardiomyocytes survival. 

Recent research has shown a close association between ER stress, Ca^2+^ signalling, STAT3 activation and Zn^2+^ homeostasis in I/R cardioprotection (Figure 1). Zinc deficiency triggers ER stress and enhances RyR2 expression in the ER, thus, mediating Ca^2+^ release and Ca^2+^-calmodulin-dependent protein kinase (CaMKII) phosphorylation. Subsequently, phosphorylated CaMKII activates STAT3, which promotes *ZIP9* expression to normalise the imbalance in Zn^2+^ homeostasis [83]. CaMKII is a serine/threonine-specific protein kinase that is regulated by the Ca^2+^/calmodulin complex, mediating many of the second messenger effects of Ca^2+^, and is involved in the regulation of many cellular and developmental processes, including cardiac rhythm [84]. Further, researchers have confirmed that reperfusion can initiate ER stress and cause an increase in intracellular Ca^2+^ and CaMKII activation [85].

Interestingly, a ZIP13 zinc transporter was shown to modulate the Ca^2+^ signalling in myocardial I/R injury. In particular, heart-specific knockout of ZIP13 leads to CaMKII activation, mitochondrial swelling, increases the mitochondrial Ca^2+^, ROS production, decreases the mitochondrial respiration control rate and dissipation of mitochondrial membrane potential in a CaMKII-dependent manner, and finally, exacerbates myocardial infarction in mouse hearts subjected to I/R. On the contrary, *ZIP13* overexpression suppresses I/R-induced CaMKII phosphorylation and reduces infarct size [86].

These data suggest the involvement of Zn^2+^ in the functioning of a new ER stress/CaMKII/STAT3 cardioprotective mechanism, activated in the heart to increase the resistance to I/R injury. Furthermore, the interplay of Zn^2+^ with ER stress/CaMKII/STAT3 axis provides new pharmacological and therapeutic targets for treating diseases associated with zinc deficiency.

## 4. Aged Heart

Ageing is the dominant risk factor for cardiovascular diseases, as it contributes to cardiac morbidity and mortality in the aged population. The pathogenesis of cardiac ageing is associated with several parameters: dysregulation in Ca^2+^ homeostasis and changes in other ions exchange across sarcolemma, action potential prolongation, increased fibrosis, rate of mitochondrial defects and oxidative stress pressure in cardiomyocytes. Mechanically, mitochondrial oxidative stress and lipid overload increase sarcoplasmic reticulum Ca^2+^-leak through RyR2 channels, cause structural remodelling and affect the electrical and mechanical activation of the left ventricle [87,88]. In general, it is known that increased systematic and cardiac ROS production and oxidative stress, alongside decreased antioxidant capacity, are the main triggers of the development of aged-related insufficiencies. A comparison of aged and adult rats showed that aged rats have depressed contraction and relaxation activities in aortic rings, increased heart rate and mean arterial pressure, significantly prolonged RR and QT intervals, decreased ejection-fraction and preload-recruitable stroke work, alongside clear insulin resistance and hypertrophy in aged rats with normal fasting blood glucose. Further, an investigation of aged rats with a microscope suggested irregularly clustered mitochondria and lysosomes around the myofilaments in cardiomyocytes, flattened and partial local splitting in elastic lamellas in the aorta and increased muscle fibre radius and amount of collagen fibres in the heart [89,90]. Zinc is crucial for many cellular processes, including memory formation, immune function, reproduction, and antioxidant capacity. However, both zinc overload and deficiency are associated with cellular dysfunction and disease. Zinc deficiency is associated with premature ageing and the development of age-related diseases. On the contrary, zinc supplementation was shown to decrease markers of oxidative stress and the production of pro-inflammatory cytokines in elderly patients. However, surplus zinc supplementation induces senescence of several cell types and reduces lifespan in *Caenorhabditis elegans* [91,92]. Further in this section, we focus on recent publications investigating the role of zinc in ageing-related functional and structural changes in the cardiovascular system. 

It is known that some zinc transporters are upregulated by pro-inflammatory stimuli, such as IL-6 (interleukin 6), in an age-dependent manner. Ageing is responsible for a significant increase in serum IL-6 and tissue-specific increases in Zn^2+^ concentration of skeletal muscle and white adipose tissue. Additionally, the proper functioning of ZIP8 and ZIP14 is necessary to maintain trabecular and cortical bone density during ageing, and could influence the development of such diseases as sarcopenia and osteoporosis [93].

**Figure 2:**
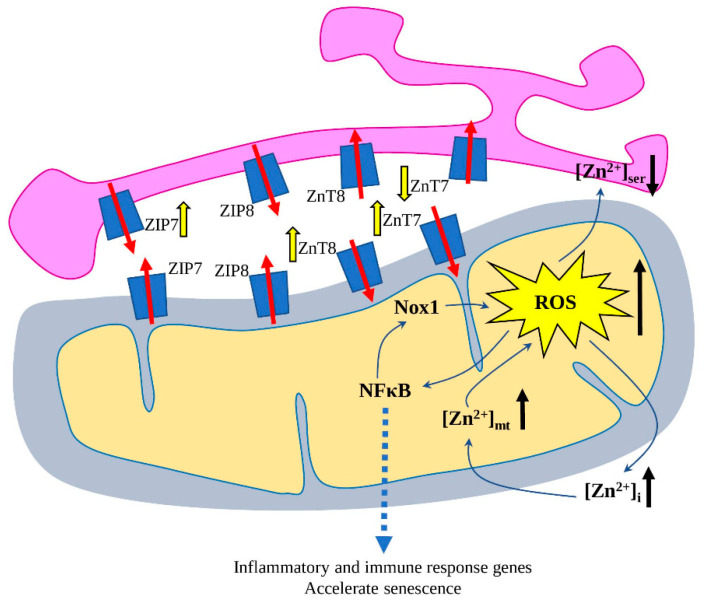
Redistribution of [Zn^2+^]_i_, [Zn^2+^]_mt_ and [Zn^2+^]_ser_ in the aged cardiomyocytes during the development of ageing-mediated cardiac dysfunction. The increased ROS production is one of the leading factors, leading to the redistribution of Zn^2+^ between cytosol, mitochondria and sarco/endoplasmic reticulum (SER) via modulated expression of Zn^2+^ transporters. ROS activates nuclear factor-κ B (NFκB), which translocates to the nucleus and activates different genes related to immune and inflammatory response. Further, NFκB increases NADPH Oxidase 1 (*Nox1*) expression, thus, further increasing ROS production and resulting in accelerated senescence. The ROS-mediated effects on the Zn^2+^ transporters are depicted with yellow arrows; the direction of Zn^2+^ transport—with red arrows, increased/decreased level of ROS and Zn^2+^—with black arrows.

Aged cardiomyocytes had an increased [Zn^2+^]_i_ level due to decreased levels of ZIP8 and ZnT7, and increased ZIP7 and ZnT8 transporters (Figure 2). Similarly, aged H9c2 cells demonstrated a significant increase in mitochondrial Zn^2+^ ([Zn^2+^]_mt_) with decreases in sarco/endoplasmic reticulum Zn^2+^ ([Zn^2+^]_ser_), where expression of mitochondrial Zn^2+^ transporters *ZNT7* and *ZNT8* was increased, and in the case of S/ER Zn^2+^ transporters, decreased expression of *ZNT7* and increased *ZIP7* [94].

Zinc overload increases the production of mitochondrial ROS, activates NFκB and contributes to the increase in NADPH Oxidase 1 (*Nox1*) expression and stimulates senescence vascular smooth muscle cells (VSMCs) (Figure 2) [95]. NFκB is a crucial transcription regulator, which is activated by various stimuli (such as cytokines, oxidant-free radicals, pathogens), translocates into the nucleus and stimulates the expression of a wide variety of inflammatory and immune-response-related genes [96,97]. Mechanically, Zn^2+^ acts via ZnT3 and ZnT10 transporters, which are known to reduce cytosolic zinc. Interestingly, other metals (copper, iron, cobalt and manganese) did not affect *NOX1* expression, suggesting its zinc-specific upregulation. Further, Nox1 induces VSMCs’ senescence by both telomere-dependent and -independent pathways, which was also associated with increased DNA damage and reduced proliferation [95].

In total, the presented data demonstrated the crucial role of precise Zn^2+^ distribution among cytosol and organelles in heart ageing. Ageing-associated dysregulation of Zn^2+^ transporters leads to intracellular and mitochondrial Zn^2+^ overload, increased ROS production and accelerated senescence. However, application of direct mitochondria-targeting antioxidant treatment can be an effective therapeutic strategy to overcome the ageing-associated Zn^2+^-mediated rise of mitochondrial ROS and cardiomyocyte dysfunction. Further, target pharmacological inhibition/stimulation of the Zn^2+^ transporters, responsible for the Zn^2+^ redistribution among suborganelles in aged cardiomyocytes, could be another potential cardioprotective approach against ageing-associated cardiac dysfunction.

## 5. Conclusions

Zn^2+^ plays a vital role in normal cell physiology and in pathophysiological conditions, such as cardiovascular diseases, premature ageing and ageing-associated diseases, diabetes and insulin resistance. Zn^2+^ uptake, influx/efflux, distribution and storage in organelles are tightly controlled by many signalling pathways and associated with the homeostasis of the other two valent ions (primarily Ca^2+^). Both surplus zinc supply and deficiency have been associated with a number of cardiovascular diseases, cardiac developmental abnormalities and accelerated cardiac ageing. In this review, we documented the role of Zn^2+^ for mitochondrial function, heart development, electrical and mechanical functions, heart failure and ischemia/reperfusion injury. Because mitochondria are the primary source of ROS responsible for the induction and progression of many cardiovascular diseases and heart ageing, the application of pharmacological agents targeting mitochondrial Zn^2+^-transporters to normalise [Zn^2+^]_mt_ and ROS levels could be an effective strategy for the prevention and/or therapy of cardiovascular dysfunction in humans. 

## Data Availability

Not applicable.

## Abbreviations

[Zn^2+^]_i_	intracellular labile Zn^2+^
[Zn^2+^]_mt_	mitochondrial Zn^2+^
[Zn^2+^]_ser_	sarco/endoplasmic reticulum Zn^2+^
ADAMTS	A Disintegrin And Metalloproteinase With Thrombospondin Motifs 1
BAX	B-cell lymphoma 2–associated X protein
BBC3	BCL2 Binding Component 3
Beclin1	ATG6, Autophagy Related
calregulin	Endoplasmic Reticulum Resident Protein 60
CaMKII	Ca^2+^-calmodulin-dependent protein kinase
CASP	caspase 9
CDKN1A	cyclin-dependent kinase inhibitor 1A
CHD	congenital heart disease
DRP1	DNM1L or dynamin 1 like
ECM	extracellular matrix
EI24	etoposide-induced 2.4
ER	endoplasmic reticulum
ErbB2	Erb-B2 Receptor Tyrosine Kinase 2
ETC	electron transport chain
FAS	Fas cell-surface death receptor
GRP78	Heat Shock Protein 70 Family Protein 5
GSK3B	Glycogen Synthase Kinase-3 Beta
H/R	hypoxia-oxygenation
HF	Heart failure
I/R	ischemia/reperfusion injury
IκBα	NF-Kappa-B Inhibitor Alpha
LVNC	left ventricular noncompaction
MAPK1	Mitogen-Activated Protein Kinase 1, or ERK
MG23	transmembrane protein mitsugumin 23
MMP	mitochondrial membrane potential
mPTP	mitochondrial permeability transition pore
MTF1	metal regulatory transcription factor 1
NOX2	NADPH oxidase 2
RNS	reactive nitrogen species
ROS	reactive oxygen species
SENP5	SUMO1/Sentrin Specific Peptidase 5
SR	sarcoplasmic reticulum
ZIP	Zrt-/Irt-like protein
ZnT	zinc transporters
Znt9	SLC-30A9, Solute Carrier Family 30 Member 9
